# Antagonism of Bacteria from Dog Dental Plaque against Human Cariogenic Bacteria

**DOI:** 10.1155/2018/2780948

**Published:** 2018-11-04

**Authors:** Káthia Santana Martins, Lorena Tirza de Assis Magalhães, Jeferson Geison de Almeida, Fábio Alessandro Pieri

**Affiliations:** Laboratory of Microbiology Studies, Life Sciences Institute, Campus Governador Valadares, Federal University of Juiz de Fora, Rua São Paulo 745, Centro, Governador Valadares, MG, 35010180, Brazil

## Abstract

Dental caries are a process of demineralization and destruction of human teeth. They originate through many factors and are associated with biofilm formation, which consists of bacteria adhered to the teeth that form a structurally and functionally organized mass called dental plaque. Both the presence of* Streptococcus mutans* and the frequent consumption of sucrose correlate with a higher prevalence of caries in humans. In dogs, however, the incidence of this disease is low, due to factors such as differences in dental microbiota and/or their low consumption of sucrose. This work evaluated the antagonism of bacteria from dog's dental plaque against* S. mutans*, for the identification of producing strains of biotechnological products for use in preventing caries. This study used 95 bacterial isolates of canine dental plaque from the Veterinary Department at the Federal University of Viçosa, Minas Gerais, Brazil. A spot-on-the-lawn method was performed using Brain Heart Infusion agar with catalase for an initial identification of the antagonistic activity. Additional tests were conducted on the isolates classified as antagonists for confirmation of the activity, using modified Mann-Rogosa-Sharpe medium containing low dextrose concentration. These isolates were incubated at 37°C for 24 hours in anaerobiosis. The peptide nature of inhibition was evaluated using the following proteinases: proteinase K from* Tritirachium album*, bovine pancreatic trypsin, and type XII-A *α*-amylase from* Bacillus licheniformis*. In the initial identification of those strains exhibiting antimicrobial activity, 14 were classified as antagonists. One of the isolates (*Bacillus *sp.) indicated bacteriocinogenic activity, with a deformed inhibition halo on* S. mutans* by the addition of trypsin. These results suggest that this bacterial isolate may be applicable to biotechnological use to combat the main etiological agent of caries in humans. Further studies are needed to evaluate the bacteriocinogenic nature of the antimicrobial activities of the other 13 antagonistic bacterial isolates.

## 1. Introduction

Dental caries is a multifactorial disease, where microbial involvement and the host response are both of fundamental importance. The genesis of caries is associated with the formation of a biofilm consisting of bacteria that adhere to the surface of the tooth. These bacteria form a structurally and functionally organized mass called dental plaque [1–4]. Caries is the result of a chronic process that, according to Newbrun [5], appears after the interaction and presence of four factors: a susceptible teeth, microorganisms, diet, and time [6]. The aetiology of caries disease involves* Streptococcus mutans*, an acidophilic and acidogenic microorganism important in the production of acid in human dental plaque [7, 8]. The presence of* S. mutans* and the frequent consumption of sugars are directly correlated with a higher prevalence and incidence of caries [9].

In dogs, dental caries are somewhat unusual. The differences in a dog's dental microbiota, their poor sucrose consumption [10, 11], or the possibility of microorganisms present in the oral cavity could establish antagonism on* S. mutans* and other cariogenic bacteria. This antagonism results from competition for nutrients or production of compounds inhibitory to these bacteria, bacteriocins, which would develop a critical barrier against colonization by pathogenic species [1]. Pieri and colleagues [12] investigated the microbiota of the dental plaque of dogs through isolating and identifying its bacterial components. They found that one of the most present genera in dog plaque is* Streptococcus*; however, none of the isolates presented genetic similarity of the 16S rDNA gene with* Streptococcus mutans*, indicating the absence of this species in dogs [12–14].

Bacteriocins are peptides produced by bacteria to give them a competitive advantage [1].* S. mutans* may produce bacteriocins to antagonise other bacteria in the dental plaque of humans. The species of* Streptococcus* present in the dental plaque of dogs could perform this same mechanism against* S. mutans* in the dental plaque of humans [1, 12]. Numerous lactic acid bacteria are consistently found in the dental plaque of dogs; many of these have the capacity to produce bacteriocins against similar bacteria in order to establish their colonization sites [7–9]. This ability to inhibit target strains is potentially useful in food preservation and the production of alternative antimicrobial therapeutic agents for diseased sites [15–18].

The most effective way to prevent caries is the mechanical removal of biofilm by brushing and using dental floss. However, this physical removal of biofilm is typically not enough to control the disease for the majority of the population. It is important to identify additive resources to combat dental biofilm, such as chlorhexidine [4, 13]. Chlorhexidine is currently considered as an antiseptic reference in dentistry and is approved by the American Dental Association Council on Dental Therapeutics. However, the continuous use of this product in the oral cavity has numerous side effects, including burning in the oral cavity, ulcerations in the jugal mucosa, darkening of the dental enamel, and loss of taste [4, 19–21]. Consequently, there has recently been increased interest in the development of new antimicrobial chemotherapeutic agents with potential for incorporation into oral products that control cariogenic microbiota [1, 4, 9, 17, 22–26].

Caries is a major public health problem, reaching between 60 and 90% of school-age children and a large majority of adults in developed and developing countries [27, 28]. The work presented here furthers the development of anticaries products through evaluating the antagonism of bacterial isolates from canine dental plaques against* S. mutans* to identify strains that produce compounds with biotechnological potential.

## 2. Materials and Methods

All methods in this study were conducted at the Laboratory of Microbiology Studies, Institute of Life Sciences, Federal University of Juiz de Fora, Campus Governador Valadares (UFJF-GV). The antagonistic potential of 95 bacterial isolates from the dental plaque of 10 dogs was previously obtained by Pieri and colleagues [12], from January to December of 2009 (Table S1). These isolates were within the bacterial repository of the Veterinary Department at the Federal University of Viçosa, and were selected representing equal percentage of its genus in total isolates obtained from the dogs.* Streptococcus mutans* (ATCC UA159) was used as cariogenic target strain.* Staphylococcus aureus* (ATCC 25923) were used as the traditional target cultures for these assays. All cultures were stored in Brain Heart Infusion (BHI) broth, with 20% glycerol as cryoprotectant, at -80°C until use. The analysis of the antagonistic activity of the isolates was performed according to Moraes and colleagues [16], by using spot-on-the-lawn methodology for initial identification of antimicrobial activity.

**Figure 1:**
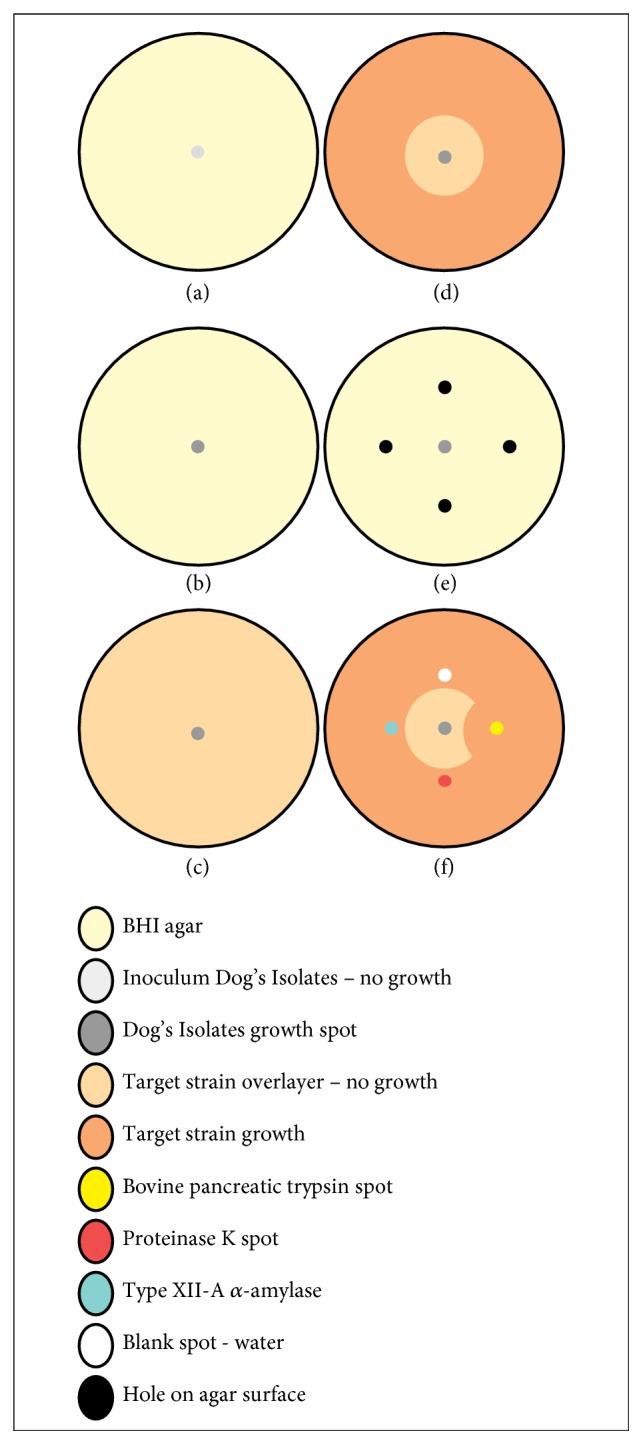
Sequence of spot-on-the-lawn surface to establish antagonistic activity of one strain of bacteria against a target strain and to confirm the protein nature of this inhibition; (a) 2 *μ*L of test strain is inoculated on the surface of Petri dishes containing agar; (b) formation of the colonies of test strain on the agar surface; (c) addition of a semisolid agar overlayer with the target bacteria; (d) inhibition halo after the incubation of target strain, confirming antagonistic activity; (e) four holes adjacent to the colonies made to inoculation of different proteinases; (f) interference of bovine pancreatic trypsin on inhibition halo of test strain against target strain, confirming the protein nature of antagonistic activity.

Bacterial cultures of dog plaque were activated for 24 hours at 37°C. From these cultures, 2 *μ*L were inoculated on the surface of Petri dishes containing BHI agar and a catalase solution (100 IU/mL) and subsequently incubated at 37°C for 24 hours [29, 30] (Figure 1(a)). After the formation of the colonies on the agar surface (Figure 1(b)), a 10 mL overlay of BHI semisolid agar was added (0.75% bacteriological agar), containing approximately 106 CFU/mL of the target bacteria:* S. mutans* (ATCC UA159) and* S. aureus* (ATCC 25923) (Figure 1(c)). After solidification of the overlayer, the plates were again incubated at 37°C for 24 hours. After that incubation step, the formation of any inhibition halo around the bacterial colony indicated antagonism (Figure 1(d)). All tests were conducted in triplicate.

The cultures that presented antagonistic activity were subjected to additional tests to confirm the protein nature of the produced antimicrobial substances [16]. A modified Mann-Rogosa-Sharpe (MRS) agar plate containing dextrose at a low concentration (5 g/L) was used, where 2 *μ*L of one of the cultures of active isolates of the dog dental plaque was inoculated in the centre of the plate and incubated anaerobically at 37°C for 24 hours (Figure 1(a)). After incubation, four holes adjacent to the colonies formed (Figure 1(e)) were inoculated with 30 *μ*L of one of the following solutions: sterile distilled water (negative control),* Tritirachium album* proteinase K (Sigma-Aldrich, Saint Louis, MI, USA), bovine pancreas trypsin (Sigma-Aldrich, Saint Louis, MI, USA), and* Bacillus licheniformis* type XII-A *α*-amylase (Sigma-Aldrich, Saint Louis, MI, USA). All inoculated enzymes held concentrations of 20mg/mL. The plates were then incubated at 37°C for two hours to allow diffusion of the inoculated solutions into the agar. After diffusion, a 10 mL overlay of BHI semisolid agar, seeded with approximately one million CFU/mL of the target microorganism, was added, followed by incubation for 24 hours at 37°C. Sensitivity of the antagonistic culture to the proteolytic enzyme solutions was observed as interference in the inoculated regions (Figure 1(f)), thus confirming the protein nature of the inhibitory substance.

## 3. Results and Discussion

From the 95 isolates tested in the initial evaluation of the antagonistic activity, 14 formed inhibition halos around the colonies in the BHI agar medium containing a catalase solution. These isolates were initially classified as antagonists against* S. mutans* and/or* S. aureus*.

Of the 14 isolates initially classified as antagonists, only one did not show growth in the dextrose-modified MRS medium and was subsequently discarded. The other 13 strains showed growth and formation of inhibition halos around the colonies when incubated with the target strains. These 13 isolates which were confirmed as antagonists and the diameters of their inhibition halos were evaluated (Table 1).

Of the 13 isolates, only* Bacillus* sp. (HQ717211; Table 1) had an inhibition halo that was modified by proteolytic enzyme solution (bovine pancreas trypsin). These results indicated the sensitivity of the culture to the enzyme solution, thereby confirming the protein nature of the inhibitory substance.

The spot-on-the-lawn methodology is widely used to detect the protein character of antimicrobial substances produced from bacteria. It is considered advantageous, as proteins are distinguishable even in cultures that produce small inhibition halos [16, 30, 31]. This methodology was performed using BHI medium with catalase (100 IU/mL) incubated at 37°C for 24 hours for the initial identification of the antimicrobial activity. The catalase solution was added to hydrolyse any possible hydrogen peroxide produced by the cultures [16, 32, 33]. Hydrogen peroxide has antimicrobial potential and could thus interfere with the identification of isolates with antagonistic activity derived from the production of bacteriocins [16, 34]. The inhibition halos identified in the 14 isolates classified as antagonists had no relation to hydrogen peroxide production.

Although any one acidogenic bacterium may contribute to enamel demineralization that results in caries, the* S. mutans* strain presents additional characteristics that may initiate and exacerbate the disease [1, 35]. In addition to the fermentation of sucrose in organic acids,* S. mutans* hydrolyses substrate forming polymers, allowing them to coaggregate with other bacteria, thus forming an extracellular matrix with greater biodiversity [1, 35]. Although other human oral bacteria such as* S. sanguis*,* S. salivarius,* and* S. gordonii*, can synthesize these polysaccharides, only* S. mutans* presents a preference to the presence of sucrose in the infection site [1, 35]. In addition,* S. mutans* has the ability to store amylopectin intracellular polysaccharides for fermentation in the absence of extracellular carbohydrates, allowing continuous fermentation between host meals.* S. mutans* also shows greater release of acid when compared to other bacteria of the genus* Streptococcus* [1]. Therefore, the reduction of* S. mutans* levels in the plaque microbiota could become a desirable strategy for the prevention and treatment of the disease [9].

*Streptococcus* spp., obtained from dental plaques of dogs (supplementary data), were expected to act as potential inhibitors of* S. mutans*, as bacteriocins are produced by a microorganism to antagonise those with high genetic similarity [1, 29]. However, of the eight isolates of* Streptococcus* spp. tested in this study, none presented antagonistic activity. Our data showed a large variety of bacteria presenting antagonism against* S. mutans*. These species include* Enterococcus faecalis*,* Actinomyces* sp.,* Aerococcus viridans*,* Bacillus* sp., and* Lactococcus lactis*. Among the isolates classified at the end of the second stage of the incubation, 53.9% of the antagonists were* Enterococcus faecalis* (Table 1). This bacterial species was previously classified as* Streptococcus faecalis*, due to its high genetic and phenotypic similarity with the* Streptococcus* genus [36]. Considering that similarity, the bacteria of the genus* Enterococcus *could potentially antagonise those species within the genus* Streptococcus*.

With regard to the second phase of evaluating antagonistic activity of the bacterial isolates, the use of the MRS medium under anaerobic conditions tends to inhibit the production of hydrogen peroxide [31, 33]. In addition to the exclusion of the possible hydrogen peroxide activity on target strains, this medium and culture condition caused an increase in the diameter of the halos of some test strains, indicating antagonistic activity.

Following the aim of this work, the protein character of the antagonist activity of isolates was evaluated against* S. mutans*. The* S. aureus* overlay was used as a comparison strain in the initial phase of evaluation of the antagonistic activity, as it is used often with the spot-on-the-lawn method. However, as* S. aureus* is a recognized pathogen for both human and animal health [24], new studies evaluating the protein nature of isolates from canine dental plaque should be carried out for a possible identification of bioproducts for the treatment of diseases caused by this pathogen.

While 13 strains showed the formation of an inhibition halo in the overlay of* S. mutans*, only one isolate (HQ717211,* Bacillus* sp.) showed deformation of the halo in the deposit site of proteolytic enzyme bovine pancreatic trypsin, suggesting the loss of antagonistic activity by the interaction of the enzyme and the protein-based bacteriocin produced by this isolate.

## 4. Conclusions

This study shows that the isolate of* Bacillus* sp. (HQ717211), obtained from canine dental plaque, has biotechnological potential in combatting a major etiologic agent of caries in humans. In addition, 13 other bacterial isolates were identified as potential antagonists of* S. mutans*. Future work confirming the bacteriocinogenic nature of these isolates should be considered for use in preventive therapy and treatment of caries in humans.

## Figures and Tables

**Table 1:** Inhibition of pathogenic bacteria by bacterial isolates from dog's dental plaque. The 16S RNA Genbank access codes, genus/species, target strain, and diameter of the inhibition halo for the 13 bacterial isolates, initially classified as antagonistic against *S. mutans* and *S. aureus.*

Genbank access code	Genus/species	Target strain	Inhibition Halo (mm)
HQ717194	*Aerococcus viridans*	*S. mutans*	25
		*S. aureus*	-
HQ717297	*Lactococcus lactis*	*S. mutans*	12
		*S. aureus*	12
HQ717208	*Actinomyces *sp.	*S. mutans*	20
		*S. aureus*	-
HQ717211	*Bacillu*s sp.	*S. mutans*	22
		*S. aureus*	-
HQ717189	*Enterococcus faecalis*	*S. mutans*	23
		*S. aureus*	7
HQ717204	*Enterococcus faecalis*	*S. mutans*	18
		*S. aureus*	-
HQ717193	*Enterococcus faecalis*	*S. mutans*	17
		*S. aureus*	-
HQ717209	*Enterococcus faecalis*	*S. mutans*	22
		*S. aureus*	14
HQ717177	*Enterococcus faecalis*	*S. mutans*	22
		*S. aureus*	-
HQ717206	*Actinomyces sp.*	*S. mutans*	21
		*S. aureus*	-
HQ717183	*Enterococcus faecalis*	*S. mutans*	19
		*S. aureus*	-
HQ717203	*Enterococcus faecalis*	*S. mutans*	24
		*S. aureus*	12
HQ717328	*Actinomyces *sp.	*S. mutans*	25
		*S. aureus*	11

## Data Availability

The 16S rDNA sequences for all isolates from canine dental plaque used to identify the bacteria involved on the findings of this study have been deposited in the Genbank repository, (Genbank access codes are located in Tables 1 and S1).

## Supplementary Materials

Supplementary Materials Table S1: all isolates from dog's dental plaque used for the study of bacteriocinogenic activity against* Streptococcus mutans* and their respective Genbank access number for 16S rDNA sequences.

## References

[1] Pepperney A., Chikindas M. L. (2011). Antibacterial Peptides: Opportunities for the Prevention and Treatment of Dental Caries. *Probiotics and Antimicrobial Proteins*.

[2] Marsh P. D. (2003). Plaque as a biofilm: Pharmacological principles of drug delivery and action in the sub- and supragingival environment. *Oral Diseases*.

[3] Marsh P. D. (2006). Dental plaque as a biofilm and a microbial community - Implications for health and disease. *BMC Oral Health*.

[4] de Melo N. I., de Carvalho C. E., Fracarolli L. (2015). Antimicrobial activity of the essential oil of Tetradenia riparia (Hochst.) Codd. (Lamiaceae) against cariogenic bacteria. *Brazilian Journal of Microbiology*.

[5] Newbrun E. (1983). *Cariology*.

[6] Lima J. E. (2007). Cárie dentária: um novo conceito. *Revista Dental Press de Ortodontia e Ortopedia Facial*.

[7] Johansson E., Claesson R., van Dijken J. W. V. (2009). Antibacterial effect of ozone on cariogenic bacterial species. *Journal of Dentistry*.

[8] Li L., He J., Eckert R. (2010). Design and Characterization of an Acid-Activated Antimicrobial Peptide. *Chemical Biology & Drug Design*.

[9] Oliveira R. V., Albuquerque Y. E., Spolidorio D. M., Koga-Ito C. Y., Giro E. M., Brighenti F. L. (2016). Effect of dietary sugars on dual-species biofilms of Streptococcus mutans and Streptococcus sobrinus – a pilot study. *Revista De Odontologia Da Unesp*.

[10] Takada K., Hayashi K., Sasaki K., Sato T., Hirasawa M. (2006). Selectivity of Mitis Salivarius agar and a new selective medium for oral streptococci in dogs. *Journal of Microbiological Methods*.

[11] Elliott D. R., Wilson M., Buckley C. M. F., Spratt D. A. (2005). Cultivable oral microbiota of domestic dogs. *Journal of Clinical Microbiology*.

[12] Pieri F. A., Silva V. O., Silva-Junior A., Moreira M. A. S. (2018). Cultivable microbiota in Mitis Salivarius agar from dental plaque of dogs. *Animal and Veterinary Sciences*.

[13] Barnett M. L. (2006). The rationale for the daily use of an antimicrobial mouthrinse. *The Journal of the American Dental Association*.

[14] Riggio M. P., Lennon A., Taylor D. J., Bennett D. (2011). Molecular identification of bacteria associated with canine periodontal disease. *Veterinary Microbiology*.

[15] Wescombe P. A., Tagg J. R. (2003). Purification and characterization of streptin, a type a1 lantibiotic produced by streptococcus pyogenes. *Applied and Environmental Microbiology*.

[16] Moraes P. M., Perin L. M., Tassinari Ortolani M. B., Yamazi A. K., Viçosa G. N., Nero L. A. (2010). Protocols for the isolation and detection of lactic acid bacteria with bacteriocinogenic potential. *LWT- Food Science and Technology*.

[17] Gálvez A., Abriouel H., López R. L., Omar N. B. (2007). Bacteriocin-based strategies for food biopreservation. *International Journal of Food Microbiology*.

[18] Kyllar M., Witter K. (2005). Prevalence of dental disorders in pet dogs. *Veterinarni Medicina*.

[19] Pieri F. A. (2012). *Atividade antimicrobiana do óleo de copaíba (Copaifera langsdorffii) e seus constituintes, e avaliação do bioproduto obtido na inibição de bactérias da placa dental de cães (Doctored in Veterinary Medicine)*.

[20] Greenberg M., Dodds M., Tian M. (2008). Naturally occurring phenolic antibacterial compounds show effectiveness against oral bacteria by a quantitative structure-activity relationship study. *Journal of Agricultural and Food Chemistry*.

[21] More G., Tshikalange T. E., Lall N., Botha F., Meyer J. J. M. (2008). Antimicrobial activity of medicinal plants against oral microorganisms. *Journal of Ethnopharmacology*.

[22] Wimpenny J. W. T., Wimpenny J. (1994). The spatial organisation of biofilm. *Bacterial biofilms and their control in medicine and industry*.

[23] Palombo E. A. (2011). Traditional Medicinal Plant Extracts and Natural Products with Activity against Oral Bacteria: Potential Application in the Prevention and Treatment of Oral Diseases. *Evidence-Based Complementary and Alternative Medicine*.

[24] Carvalho S., Carmo L., Abreu E. (2013). TSST-1, enterotoxin and bacteriocin-like substance production by Staphylococcus aureus isolated from foods. *Arquivo Brasileiro de Medicina Veterinária e Zootecnia*.

[25] Pieri F. A., Silva V. O., Vargas F. S., Veiga Junior V. F., Moreira M. A. S. (2014). Antimicrobial activity of Copaifera langsdorffii oil and evaluation of its most bioactive fraction against bacteria of dog's dental plaque. *Pakistan Veterinary Journal*.

[26] Pieri F. A., Souza M. C., Vermelho L. L. (2016). Use of *β*-caryophyllene to combat bacterial dental plaque formation in dogs. *BMC Veterinary Research*.

[27] Petersen P. E. (2003). The World Oral Health Report 2003: continuous improvement of oral health in the 21st century—the approach of the WHO Global Oral Health Programme. *Community Dentistry and Oral Epidemiology*.

[28] Rocha N. B. *Condições de saúde bucal e características sócio-comportamentais de gestantes influenciam o desenvolvimento e experiência de cárie em crianças de 4 anos? (online), SciELOemPerspectiva | Press Releases*.

[29] Tagg J. R., Dajani A. S., Wannamaker L. W. (1976). Bacteriocins of gram positive bacteria. *Bacteriological Reviews*.

[30] Lewus C. B., Kaiser A., Montville T. J. (1991). Inhibition of food-borne bacterial pathogens by bacteriocins from lactic acid bacteria isolated from meat. *Applied and Environmental Microbiology*.

[31] De Martinis E. C. P., Públio M. R. P., Santarosa P. R., Freitas F. Z. (2001). Antilisterial activity of lactic acid bacteria isolated from vacuum-packaged Brazilian meat and meat products. *Brazilian Journal of Microbiology*.

[32] Moreno I., Lerayer A. L. S., De Freitas Leitão M. F. (1999). Detection and characterization of bacteriocin-producing Lactococcus lactis strains. *Brazilian Journal of Microbiology*.

[33] Schillinger U., Lücke F. K. (1989). Antibacterial activity of *Lactobacillus sake* isolated from meat. *Applied and Environmental Microbiology*.

[34] Carr F. J., Chill D., Maida N. (2002). The lactic acid bacteria: A literature survey. *Critical Reviews in Microbiology*.

[35] Kuramitsu H. K. (1993). Virulence factors of mutans streptococci: Role of molecular genetics. *Critical Reviews in Oral Biology and Medicine*.

[36] Schleifer K. H., Kilpper-Bälz R. (1984). Transfer of *Streptococcus faecalis* and *Streptococcus faecium* to the genus *Enterococcus* nom. rev. as *Enterococcus faecalis* comb. nov. and *Enterococcus faecium* comb. nov.. *International Journal of Systematic Bacteriology*.

