# Supplementary material for: Antagonism of Bacteria from Dog Dental Plaque against Human Cariogenic Bacteria
Source: Biomed Res Int. 2018 Nov 4;2018:2780948. doi: 10.1155/2018/2780948 (PMC6241363; doi:10.1155/2018/2780948)
Supplement: Supplementary Materials — Table S1: all isolates from dog's dental plaque used for the study of bacteriocinogenic activity against Streptococcus mutans and their respective Genbank access number for 16S rDNA sequences. [file 2780948.f1.docx]

**SUPPLEMENTARY MATERIAL**

## Table S1 - All isolates from dogs dental plaque used for the study of bacteriocinogenic activity against *Streptococcus mutans* and its respective genbank access number for 16S rDNA sequences.

| **N°** | **GenBank Access Number** | **Identification of isolates by BLAST search** |
| --- | --- | --- |
| 1 | HQ717349 | *Staphylococcus sp.* |
| 2 | HQ717318 | *Pasteurella sp.* |
| 3 | HQ717323 | *Staphylococcus sp.* |
| 4 | HQ717287 | *Staphylococcus sp.* |
| 5 | HQ717286 | *Staphylococcus sp.* |
| 6 | HQ717202 | *Actinomyces sp.* |
| 7 | HQ717184 | *Enterococcus faecalis* |
| 8 | HQ717179 | *Enterococcus faecalis* |
| 9 | HQ717288 | *Staphylococcus sp.* |
| 10 | HQ717291 | *Staphylococcus sp.* |
| 11 | HQ717297 | *Lactococcus lactis* |
| 12 | HQ717282 | *Streptococcus sp.* |
| 13 | HQ717294 | *Aerococcus viridans* |
| 14 | HQ717191 | *Enterococcus faecalis* |
| 15 | HQ717309 | *Staphylococcus sp.* |
| 16 | HQ717296 | *Leuconostoc sp.* |
| 17 | HQ717293 | *Neisseria sp.* |
| 18 | HQ717182 | *Staphylococcus sp.* |
| 19 | HQ717317 | *Pasteurella sp.* |
| 20 | HQ717285 | *Streptococcus sp.* |
| 21 | HQ717316 | *Streptococcus sp.* |
| 22 | HQ717312 | *Bisgaard Taxon*16 |
| 23 | HQ717313 | *Bisgaard Taxon* 16 |
| 24 | HQ717207 | *Actinomyces sp.* |
| 25 | HQ717314 | *Bisgaard Taxon* 16 |
| 26 | HQ717298 | *Neisseria sp.* |
| 27 | HQ717199 | *Actinomyces sp.* |
| 28 | HQ717213 | *Bacillus sp.* |
| 29 | HQ717175 | *Enterococcus faecalis* |
| 30 | HQ717201 | *Actinomyces sp.* |
| 31 | HQ717208 | *Actinomyces sp.* |
| 32 | HQ717196 | *Bacillus sp.* |
| 33 | HQ717211 | *Bacillus sp.* |
| 34 | HQ717192 | *Staphylococcus sp.* |
| 35 | HQ717214 | *Bacillus sp.* |
| 36 | HQ717189 | *Enterococcus faecalis* |
| 37 | HQ717176 | *Enterococcus faecalis* |
| 38 | HQ717200 | *Actinomyces sp.* |
| 39 | HQ717198 | *Actinomyces sp.* |
| 40 | HQ717190 | *Enterococcus faecalis* |
| 41 | HQ717188 | *Enterococcus faecalis* |
| 42 | HQ717197 | *Bacillus sp.* |
| 43 | HQ717204 | *Enterococcus faecalis* |
| 44 | HQ717212 | *Bacillus sp.* |
| 45 | HQ717210 | *Enterococcus faecalis* |
| 46 | HQ717178 | *Enterococcus faecalis* |
| 47 | HQ717217 | *Enterococcus faecalis* |
| 48 | HQ717193 | *Enterococcus faecalis* |
| 49 | HQ717209 | *Enterococcus faecalis* |
| 50 | HQ717177 | *Enterococcus faecalis* |
| 51 | HQ717216 | *Bacillus sp.* |
| 52 | HQ717180 | *Enterococcus faecalis* |
| 53 | HQ717194 | *Enterococcus faecalis* |
| 54 | HQ717205 | *Enterococcus faecalis* |
| 55 | HQ717206 | *Actinomyces sp.* |
| 56 | HQ717187 | *Enterococcus faecalis* |
| 57 | HQ717183 | *Enterococcus faecalis* |
| 58 | HQ717181 | *Enterococcus faecalis* |
| 59 | HQ717195 | *Actinomyces sp.* |
| 60 | HQ717203 | *Enterococcus faecalis* |
| 61 | HQ717186 | *Enterococcus faecalis* |
| 62 | HQ717215 | *Enterococcus sp.* |
| 63 | HQ717185 | *Enterococcus faecalis* |
| 64 | HQ717325 | *Pasteurella sp.* |
| 65 | HQ717326 | *Micrococcus luteus* |
| 66 | HQ717328 | *Actinomyces sp.* |
| 67 | HQ717324 | *Arthrobacter sp.* |
| 68 | HQ717321 | *Staphylococcus sp.* |
| 69 | HQ717330 | *Lactococcus lactis* |
| 70 | HQ717329 | *Leuconostoc sp.* |
| 71 | HQ717322 | *Staphylococcus sp.* |
| 72 | HQ717307 | *Leuconostoc sp.* |
| 73 | HQ717310 | *Bisgaard Taxon 16* |
| 74 | HQ717301 | *Streptococcus sp.* |
| 75 | HQ717334 | *Lactococcus lactis* |
| 76 | HQ717327 | *Pasteurella sp.* |
| 77 | HQ717306 | *Staphylococcus sp.* |
| 78 | HQ717320 | *Staphylococcus sp.* |
| 79 | HQ717335 | *Lactococcuslactis* |
| 80 | HQ717348 | *Streptococcus minor* |
| 81 | HQ717347 | *Bisgaard Taxon*16 |
| 82 | HQ717352 | *Streptococcus minor* |
| 83 | HQ717346 | *Staphylococcus sp.* |
| 84 | HQ717345 | *Staphylococcus sp.* |
| 85 | HQ717331 | *Leuconostoc sp.* |
| 86 | HQ717350 | *Enterococcus faecalis* |
| 87 | HQ717336 | *Leuconostoc sp.* |
| 88 | HQ717295 | *Neisseria sp.* |
| 89 | HQ717299 | *Streptococcus sp.* |
| 90 | HQ717304 | *Neisseria sp.* |
| 91 | HQ717315 | *Pasteurella sp.* |
| 92 | HQ717305 | *Streptococcus sp.* |
| 93 | HQ717283 | *Staphylococcus sp.* |
| 94 | HQ717319 | *Haemophilus haemoglobinophilus* |
| 95 | HQ717308 | *Leuconostoc sp.* |

Source of isolates: Pieri FA, Silva VO, Silva Junior A, Moreira, MAS. Cultivable Microbiota in Mitis Salivarius Agar from Dental Plaque of Dogs. Animal Vet Sci. 2018; 6: in press.
